# Application of Metagenomic Next-Generation Sequencing (mNGS) Using Bronchoalveolar Lavage Fluid (BALF) in Diagnosing Pneumonia of Children

**DOI:** 10.1128/spectrum.01488-22

**Published:** 2022-09-28

**Authors:** Aimei Yang, Chen Chen, Yan Hu, Guilang Zheng, Peiling Chen, Zhiwei Xie, Huifeng Fan, Yueyu Sun, Peiqiong Wu, Wenhui Jiang, Chun Wang, Jingwen Zhang, Dongwei Zhang, Jing Wang, Xiaoyin Hu, Han Xia, Genquan Yin, Yuxiong Guo

**Affiliations:** a Department of Pediatric Intensive Care Unit, Guangdong Provincial People’s Hospital, Guangdong Academy of Medical Sciences, Guangzhou, China; b Pediatric Respiratory Department, Guangzhou Women and Children's Medical Center, Guangzhou Medical University, Guangzhou, China; c Department of Scientific Affairs, Hugobiotech Co., Ltd., Beijing, China; University of Cincinnati

**Keywords:** metagenomic next-generation sequencing (mNGS), bronchoalveolar lavage fluid (BALF), pneumonia, children, culture, diagnosis

## Abstract

Pneumonia is the leading cause of death in children; the pathogens are often difficult to diagnose. In this study, the performance of metagenomic next-generation sequencing (mNGS) using bronchoalveolar lavage fluid (BALF) samples from 112 children with confirmed pneumonia has been evaluated. mNGS performed a significantly higher positive detection rate (91.07%, 95% confidence interval [CI] 83.80% to 95.40%) and coincidence rate against the final diagnosis (72.32%, 95% CI 62.93% to 80.15%) than that of conventional methods (70.54%, 95% CI 61.06% to 78.58% and 56.25%, 95% CI 46.57% to 65.50%, respectively) (*P* < 0.01 and *P* < 0.05, respectively). Bacteria, viruses, and their mixed infections were common in children with pneumonia. Streptococcus pneumoniae was the most common bacterial pathogen in children with pneumonia, while Haemophilus parainfluenzae and Haemophilus influenzae seemed more likely to cause nonsevere pneumonia in children. In contrast, human cytomegalovirus (CMV) infection and the simultaneous bacterial infections could cause severe pneumonia, especially in children with underlying diseases. After adjustments of antibiotics based on mNGS and conventional methods, the conditions improved in 109 (97.32%) children. mNGS of BALF samples has shown great advantages in diagnosing the pathogenic etiology of pneumonia in children, especially when considering the limited volumes of BALF and the previous use of empirical antibiotics, contributing to the timely adjustment of antibiotic treatments, which can potentially improve the prognosis and decrease the mortality.

**IMPORTANCE** Our study indicates high efficiency of mNGS using BALF for the detection of causative pathogens that cause pneumonia in children. mNGS can be a potential diagnostic tool to supplement conventional methods for children’s pneumonia.

## INTRODUCTION

Pneumonia is the most important cause of death in children after preterm birth complications, even before the COVID-19 outbreak (1). As estimated by the World Health Organization (WHO), approximately 0.74 million children under the age of 5 years died of lower respiratory infections in 2019 worldwide (2). Various pathogens, including bacteria, viruses, fungi, and other microorganisms, have been reported as responsible for pneumonia (3, 4). Due to the varied clinical symptoms in different patients and the diverse etiologies by age (5, 6), the specific pathogen-causing pneumonia often fail to identify in many clinical practices (7, 8). Therefore, identification and characterization of pathogenic microorganisms are crucial for precise treatment, improving prognosis, and reducing mortality in patients with pneumonia (9 to 11).

Bronchoalveolar lavage (BAL), as a standard diagnostic procedure for pulmonary diseases, has been widely applied in the diagnosis of pneumonia (12). BAL fluid (BALF) obtained from the locus of infection through BAL is a favorable specimen for the analysis of immune cells, inflammatory cells, cytology, and infectious microbial etiology at the alveolar level, assisting in the pathogen diagnosis and prognosis judgment of pneumonia (13). Other samples, such as sputum and blood, are also chosen as alternative samples for the diagnosis of pneumonia in children, but the accuracy is relatively lower than that of BALF (14 to 16). Currently, traditional etiological detection methods, such as culture, immunological assays, and PCR, are commonly applied for clinical diagnosis of pneumonia (17). However, their applications are restricted because of poor timeliness, low pathogen coverage, and insufficient rates of positive detection (17, 18). Thus, considering the complexity of etiology and unsatisfactory pathogen detection methods in patients with pneumonia, highly sensitive and accurate multiplexed diagnostic methods are necessary.

Nowadays, metagenomic next-generation sequencing (mNGS) has been increasingly used in the clinical diagnosis of many infectious diseases (19, 20). It is an unbiased approach that can rapidly detect almost all pathogens from the clinical samples with a high sensitivity and accuracy, especially for the diagnosis of rare, novel, unknown pathogens, and those with atypical etiology of complicated infectious diseases (21). Moreover, the demand for low amounts of DNA from samples has also accelerated the clinical application of mNGS in pathogen diagnosis. However, few studies have investigated the application of mNGS in diagnosing pneumonia of children using BALF samples with a large sample size.

In this study, a multicenter prospective study enrolling a total of 112 children has been performed. After BAL examination, BALF was collected for mNGS and conventional diagnostic methods detection. Furthermore, the diagnostic performance of mNGS using BALF samples in children with pneumonia was evaluated by comparing with conventional diagnostic methods.

## RESULTS

### General characteristics.

**FIG 1:**
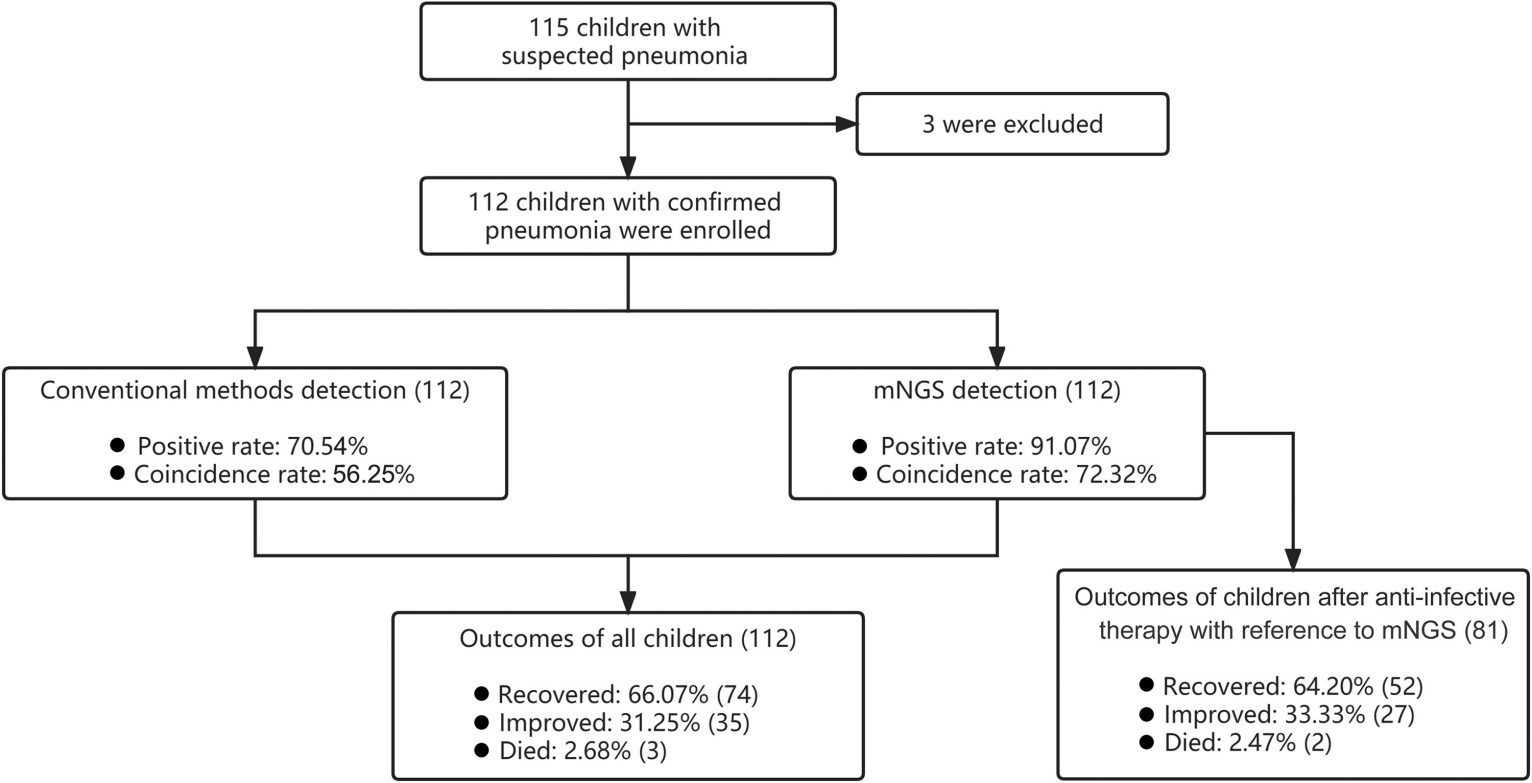
The study design, detection results of conventional methods and mNGS, and outcomes after antibiotic treatments of children with pneumonia in this study. A total of 112 children with pneumonia were enrolled in this study. The positive rates of mNGS using BALF samples and conventional methods using BALF and sputum samples were 91.07% and 70.53%, respectively. The coincidence rates against the final diagnosis by mNGS and conventional methods was 72.32% and 65.18%, respectively. After antibiotic treatments, 97.32% (109/112) patients had improved conditions.

A total of 115 children with suspected pneumonia were screened in this prospective analysis. After exclusion of 3 cases with bronchitis or upper respiratory tract infection, 112 children were included for the following analysis (Fig. 1). Out of the 112 patients, there were 67 males and 45 females. The average age of these enrolled children was 3.12 years old, ranging from 0.1 to 13 years old. Their average hospital stay was 19 days, ranging from 1 to 64 days. According to the Chinese Guidelines for Management of Community Acquired Pneumonia in Children (the revised edition of 2013) (22), 59 (52.68%) cases were diagnosed with severe pneumonia, and 57 (50.89%) were admitted to the pediatric intensive care unit (PICU) with an average ICU duration of 12 days. Thirty-two out of the 112 patients (28.57%) received mechanical ventilation with an average mechanical ventilation duration of 6 days. A total of 65 patients had underlying diseases, of whom 33 were finally diagnosed with severe pneumonia (33/65, 50.77%). The underlying diseases included congenital or other heart diseases (19), premature birth (16), recurrent respiratory infections (11), immunodeficiency disease (9), growth retardation (7), malnutrition (6), chromosomal abnormalities (4), neonatal respiratory distress syndrome (NRDS) (3), anemia (2), Down syndrome (2), Piro syndrome (2), bloodthirsty syndrome (1), congenital laryngomalacia (1), connective tissue disease (1), emphysema (1), hypertension (1), and rheumatoid arthritis (1). Multiple underlying diseases in one child was common. The outcomes of each child after antibiotic treatments were evaluated based on the clinical symptoms (such as fever, cough, congestion, shortness of breath, expectoration production, respiratory rate, lung rales, and general appearance), lung imaging, inflammatory indicators (such as leukocyte count and C-reactive protein [CRP] level), oxygen saturation, and other indicators detected by laboratory tests and conventional diagnostic methods (23). Seventy-four children were cured, and 35 children experienced improved conditions. Three children died due to severe pneumonia and congenital heart disease in this study, indicating a mortality of 2.68%. The detailed baseline of enrolled children is shown in Table 1.

**TABLE 1:** The baseline of 112 children with pneumonia enrolled in this study

Characteristic	Clinical value
Age (avg, mo)	3.12
Sex	
Male	59.8% (67/112)
Female	40.2% (45/112)
Diagnostic results	
Severe pneumonia	52.7% (59/112)
Nonsevere pneumonia	47.3% (53/112)
Other diseases	
Underlying diseases	58.0% (65/112)
Without underlying diseases	42.0% (47/112)
Admitted to the PICU	
Yes	50.9% (57/112)
No	49.1% (55/112)
Mechanical ventilation	
Yes	28.6% (32/112)
No	71.4% (55/112)
Outcomes	
Cured	66.1% (74/112)
Improved	31.3% (35/112)
Died	2.7% (3/112)

### Diagnostic performance of mNGS and conventional methods.

**FIG 2:**
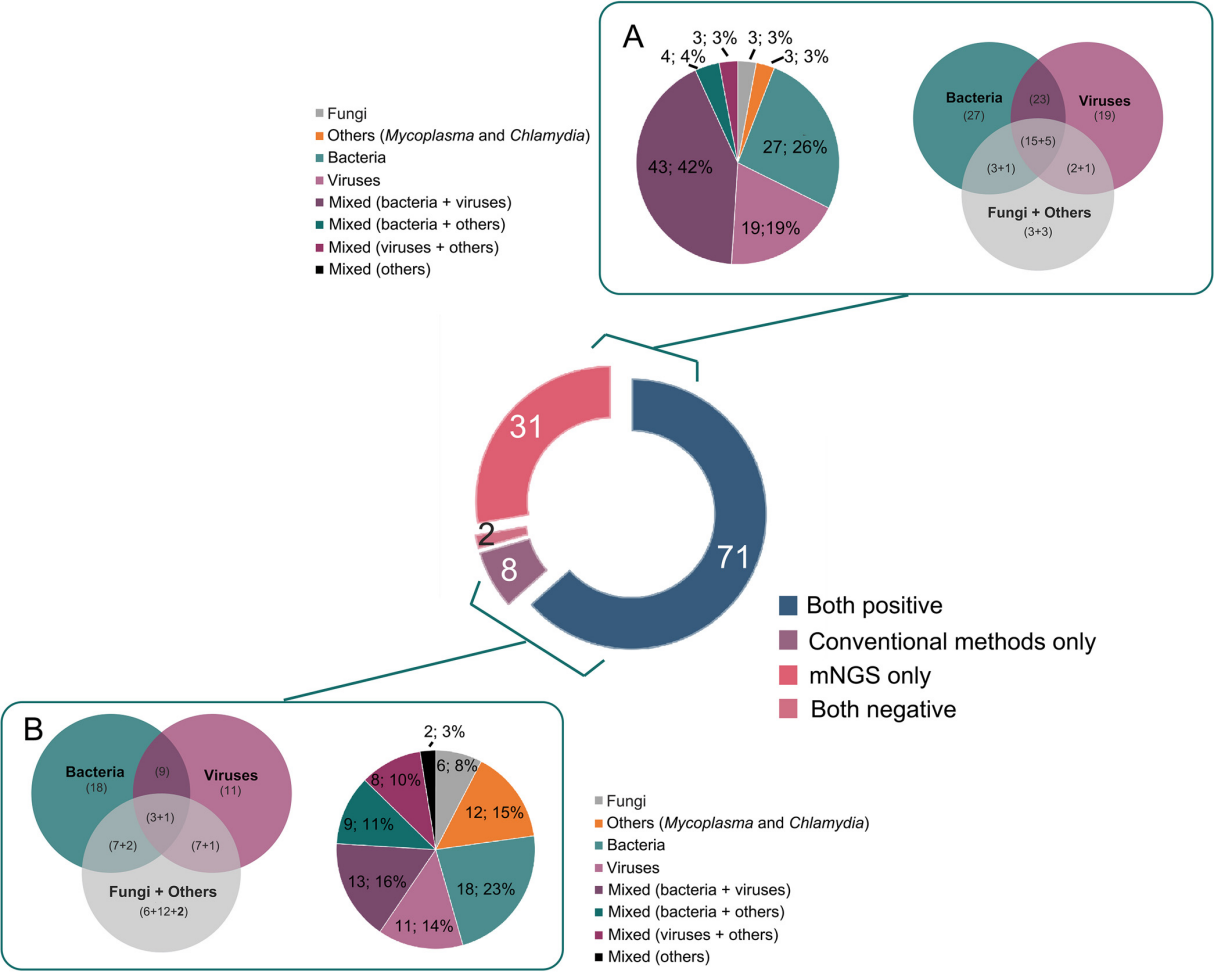
The pathogens spectrum by mNGS and conventional methods in all 112 enrolled cases. (A) Pathogen profiles detected by mNGS. (B) Pathogen profiles detected by conventional methods.

**FIG 3:**
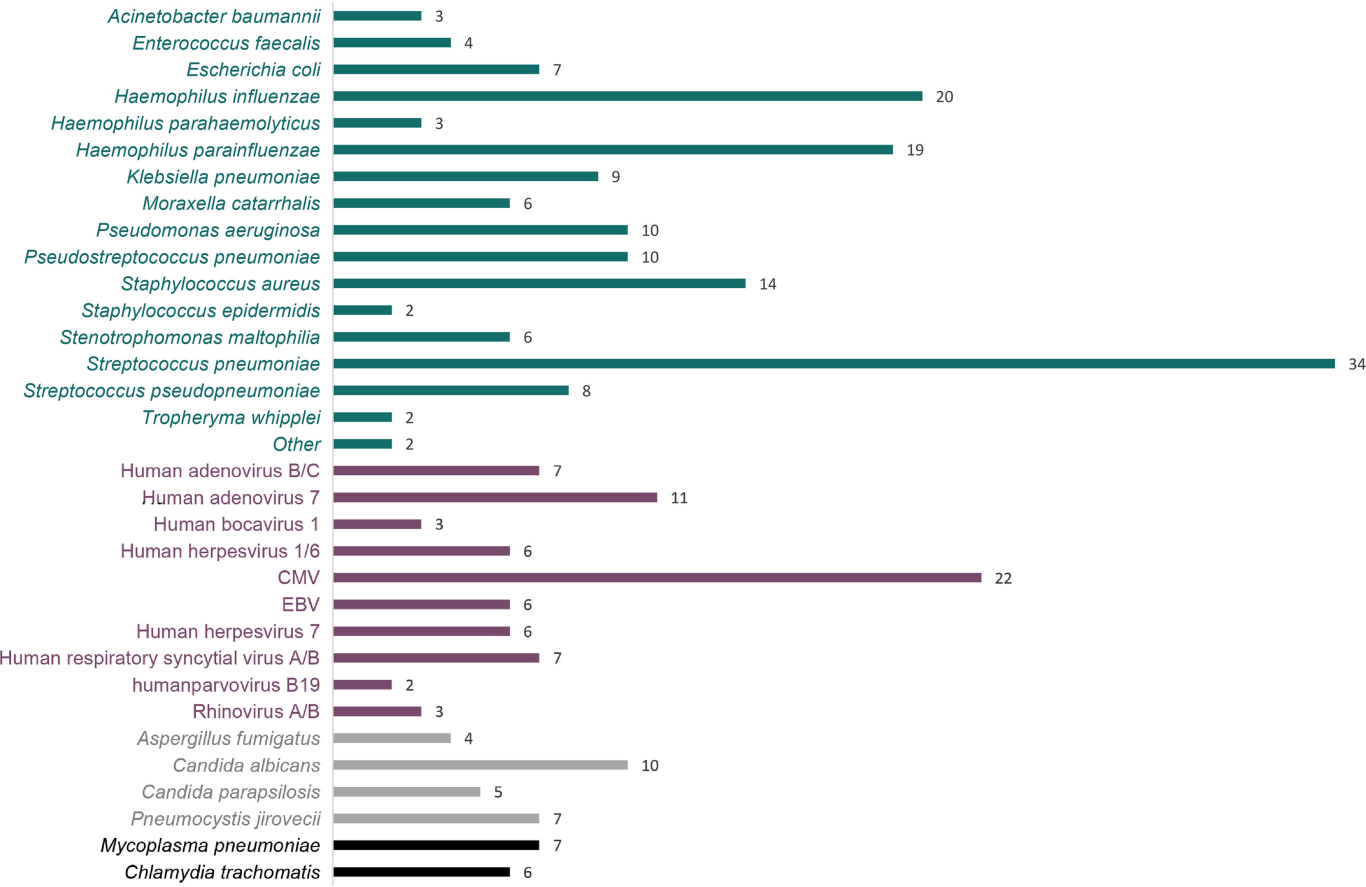
The number of pathogens of all cases detected by mNGS. Streptococcus pneumoniae, Haemophilus parainfluenzae, and Haemophilus influenzae were the leading bacterial pathogens, and human herpesvirus, human adenovirus, and human respiratory syncytial virus were the leading viral pathogens, especially for human cytomegalovirus (CMV) and human adenovirus type 7 (HAdV-7). Other pathogens, such as Candida albicans, Pneumocystis jirovecii, and Mycoplasma pneumoniae were also common in this study.

BALF samples from the 112 enrolled children with pneumonia were collected for mNGS detection, and 102 cases showed positive mNGS results, indicating a positive detection rate of 91.07% (102/112, 95% CI 83.80% to 95.40%). Pathogenic species detected in these cases consisted of 29 bacteria, 11 viruses, 8 fungi, and 2 other prokaryotes. Bacteria (26%, 27/102), viruses (19%, 19/102), and their mixed infections (42%, 43/102) were the most common in children with pneumonia based on mNGS results (Fig. 2A). The pathogen spectrum revealed that Streptococcus pneumoniae (34), Haemophilus influenzae (20), and Haemophilus parainfluenzae (19) were the leading bacterial pathogens, while human herpesvirus (39) and human adenovirus (16) were the dominating viral pathogens, especially for human cytomegalovirus (CMV) and human adenovirus type 7 (HAdV-7). Other pathogens, such as Candida albicans (10), Pneumocystis jirovecii (7), and Mycoplasma pneumoniae (7), were also frequent in this study. The detailed information of detected pathogens by mNGS is shown in Fig. 3 and Table S1.

Conventional methods were also applied in all cases, and 79 cases were positive combined with all results of conventional methods (Table S1), indicating a positive rate of 70.54% (79/112, 95% CI 61.06% to 78.58%), which was significantly lower than that of mNGS (*P < *0.01). Unlike mNGS, simple bacterial infections (23%, 18/79), simple viral infections (14%, 11/79), simple *Mycoplasma* infections (14%, 11/79), and mixed infections by bacteria and viruses (16%, 13/79) were common in children with pneumonia based on conventional methods (Fig. 2B). Besides, the results of mNGS and conventional methods were assessed against the final clinical diagnoses by two experienced clinicians based on multiple clinical factors, including the epidemiology, clinical manifestations, laboratory test results, imaging results, and outcomes after anti-infective treatments. When the final diagnosis result was consistent with certain pathogens detected by mNGS or conventional methods, a “coincidence” of the method was given. The coincidence rate of mNGS and conventional methods with the final clinical diagnoses was 72.32% (81/112, 95% CI 62.93% to 80.15%) and 56.25% (63/112, 95% CI 46.57% to 65.50%), respectively, revealing that mNGS had a significantly higher coincidence rate than conventional methods (*P < *0.05).

Considering that culture is the gold standard method for bacterial and fungal pathogens, we also compared the diagnostic results of mNGS and culture (BALF and sputum). A total of 45 cases were detected as positive by culture, with a positive rate of 40.18% (45/112, 95% CI 31.16% to 49.88%), which is obviously lower than that of mNGS (*P < *0.01). There were 8 and 61 cases with only positive culture and only positive mNGS detection, respectively. mNGS and culture detected the same pathogens in 30 cases (28 were at genus level), accounting for 66.67%. Different pathogens were detected by mNGS and sputum culture in 8 (BALF culture was negative) of the remaining 15 cases. These data indicated the high concordance between mNGS and culture detection results based on BALF samples. In this study, 88.39% (99/112) of patients received empirical antibiotics before sample collection. No significant difference was found in the positive rate of mNGS between cases with antibiotics (91%) and without antibiotics (87%). However, the positive rate of culture between the two groups of cases was 39.39% and 57.14%, respectively. Thus, culture seemed more likely to be influenced by the empirical antibiotic treatments before sampling.

### Characteristics of pathogens in severe pneumonia children.

**FIG 4:**
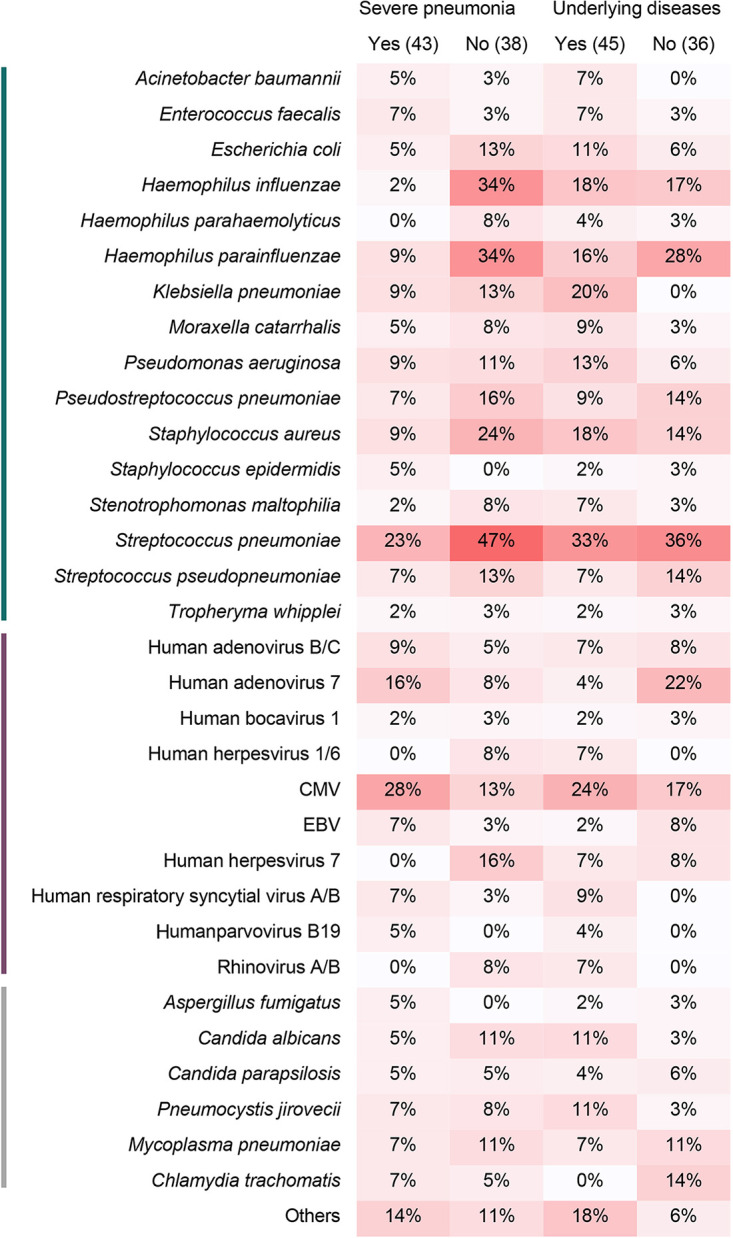
The pathogens spectrum by mNGS based on the final diagnosis between severe pneumoniae cases and nonsevere pneumoniae cases, as well as cases with and without underlying diseases.

There were 59 severe pneumonia children and 53 nonsevere pneumonia children in this study. The positive detection rate and coincidence rate of mNGS against the final clinical diagnoses in severe pneumonia children was 88.14% (52/59, 95% CI 76.46% to 94.70%) and 72.88% (43/59, 95% CI 59.51% to 83.26%), respectively, similar to that in nonsevere pneumonia children (94.34%, 95% CI 83.37% to 98.53%, and 71.70%, 95% CI 57.44% to 82.80%, respectively). Both severe and nonsevere pneumonia were dominated by multipathogen infections with bacteria and viruses. Interestingly, the proportion of simple bacterial infection in severe cases was 16.95%, while that in nonsevere cases was 32.08%, and simple viral infection accounted for 27.12% in severe cases, and only 5.66% in nonsevere cases (Fig. 4).

The most common pathogens in severe patients included S. pneumoniae (10), CMV (12), and HAdV-7 (7), while in nonsevere children, S. pneumoniae (18), H. influenzae (13), *H. parainfluenzae* (13), and S. aureus (9) were the leading pathogens (Fig. 4).

### Characteristics of pathogens in pneumonia children with underlying diseases.

A total of 65 children were diagnosed with underlying diseases in this study. Similar to the mNGS results between severe and nonsevere pneumonia children, there were no significant differences in positive detection rate and coincidence rate by mNGS between pneumonia children with underlying diseases (87.69%, 95% CI 76.64% to 94.16%, and 67.69%, 95% CI 54.82% to 78.46%, respectively) and without underlying diseases (95.74%, 95% CI 84.27% to 99.26%, and 76.60%, 95% CI 61.63% to 87.21%, respectively). The proportion of mixed infections with bacteria and viruses in children with underlying diseases was higher than that of children without underlying diseases (51% and 31%, *P < *0.05).

The main pathogens that caused pneumoniae in children with underlying diseases were S. pneumoniae (15), CMV (11), and K. pneumoniae (9). Different from the other pathogens, K. pneumoniae in children without underlying diseases were rare (Fig. 4).

### Adjustment of antibiotic treatment with reference to mNGS detection.

According to the mNGS results, empirical antibiotic treatments were adjusted in 35.7% of patients (40/112), including 28 cases with severe pneumonia, while 36.6% (41/112) of cases abandoned adjustment due to the coincidence of previous antibiotics with mNGS detection. The adjustment of antibiotics included dose reduction in 9 cases, dose increase in 19 cases, antibiotics change in 11 cases, and discontinuation in 1 case. Of the 81 children, 52 were cured, and the conditions of 27 patients improved (95.74%). The other 2 children died due to severe pneumonia and congenital heart disease.

## DISCUSSION

BALF is usually used for multiple diagnostic tests to identify pathogens causing pneumonia, which performs a higher accuracy than other types of samples, including sputum and blood (14 to 16). In this study, we successfully collected BALF from 112 children with pneumonia. Following the simultaneous pathogen detection of mNGS and conventional methods, as well as the timely adjustment of empirical antibiotics, 74 children were cured and 35 were with improved conditions. Gratifyingly, the mortality decreased from 13.5% of previous studies (24) to 2.68% (3/112) of this study based the combined diagnosis of mNGS and conventional methods.

mNGS of BALF samples and multiple conventional methods of BALF, sputum, blood, and throat swab samples were performed simultaneously in this study. Compared to the conventional methods, mNGS showed a significant advantage in detecting the causative pathogens among children with pneumonia. It is well known that BALF is difficult to collect from children, suggesting the preciousness of BALF samples. mNGS can theoretically detect all microorganisms of the sample in one run (25). In this study, only 1 mL of BALF was used for mNGS detection, and multiple pathogens, including bacteria, viruses, fungi, and other microbes (such as Mycoplasma pneumoniae and Chlamydia trachomatis), were identified in 1 to 2 days. Importantly, the coincidence rate of mNGS against the final diagnosis could reach 72.32%, significantly higher than that of overall conventional methods. Considering the difficulty of BALF collection in children and the large volume demand of samples by conventional methods, mNGS seems more suitable to detect pathogens using BALF in pneumonia children. In addition, previous studies have demonstrated that culture, as the gold standard method for bacteria and fungi detection (26), is susceptible to empirical antibiotic treatments (27). However, empirical antibiotics treatment is usually unavoidable once pneumonia patients are admitted to hospital. In this study, 88.39% of patients received empirical antibiotics before sample collection; the influence of empirical antibiotics to culture before sampling cannot be ignored. In contrast, mNGS seemed less affected by the previous antibiotic treatments. Our findings indicate that mNGS can be a supplementary diagnostic method for pneumonia in children, especially when antibiotic treatments have been used before sampling.

Pneumonia is the leading cause of death in children, with high mortality and poor prognosis, especially severe pneumonia (2, 17). In this study, we analyzed the spectrum of characteristics of pathogens in children with severe pneumonia. The results revealed that mixed infections by bacteria and viruses often occurred in not only children with severe pneumonia but also in children with nonsevere pneumonia. One reason may be that viral infections can damage the mucosal linings of respiratory tracts, which induces secondary bacterial infections (28, 29). The detailed mechanisms of coinfections by bacteria and viruses among pneumonia patients should be further explored. S. pneumoniae, S. aureus, P. aeruginosa, K. pneumoniae, and *H. parainfluenzae* were the familiar bacterial pathogens of severe pneumonia children (30). S. pneumoniae, as the most common bacterial cause of pneumonia in all age patients (31, 32), had the largest proportion (25.00%) in all pneumonia children in this study, indicating its high pathogenicity. This pathogen was also the most dominant bacterium in severe pneumonia children of the study, accounting for 44.74%. Thus, early diagnosis and timely treatment is indispensable to avoid the progression of S. pneumoniae and improve the prognosis of pneumonia patients. It should be noted that similar to the previous studies (32), only 8% (9/112) of children were diagnosed with S. pneumoniae by culture in this study, which was significantly lower than that by mNGS, indicating the underestimation of S. pneumoniae infections in clinical practice. Therefore, the addition of a superior diagnostic method such as mNGS contributes to improved clinical identification of S. pneumoniae infections.

Human herpesvirus and adenovirus were the most detected viral pathogens in this study, especially CMV and HAdV-7. HAdV-7 was more often in children with pneumonia without underlying diseases, and the simultaneous bacterial infections were rare in this study. Compared with HAdV-7, CMV and its simultaneous bacterial infections were more often in children with severe pneumonia with underlying diseases. Previous studies revealed that CMV in immunocompromised patients is common (33). In nonimmunocompromised hosts, CMV infections are considered benign and self-limited, though there are a considerable number of reports of CMV infections with severe clinical manifestations in immunocompetent patients (34). CMV is reported to be the most relevant infectious cause of permanent disabilities in children, and it can persist as a longer course of infection than in adults, playing a potential role in aggravating multiple diseases, including COVID-19 infection and colitis (35, 36). In this study, our data indicate that CMV and its simultaneous bacterial infections trend to cause severe pneumonia in children with underlying diseases. More cases are needed for further exploration.

The microbiome of the respiratory tract is complex, and many colonizing or opportunistic pathogens can cause infection, especially when the immunity of patients is weakened (37). In this study, multiple colonized microbes were detected by mNGS, such as C. albicans and H. influenzae, making it difficult to determine the causative pathogens. Multiple factors, including epidemiology, clinical manifestations, laboratory test results, imaging results, conventional diagnostic results, and outcomes after previous anti-infective treatments, were considered to help distinguish the pathogen. For example, C. albicans was detected by both mNGS and culture in patient 8, but the clinical manifestations of this child did not support C. albicans infection, indicating C. albicans in this case as a colonizer. The child was cured without antifungal treatment, confirming our diagnosis. There were no other pathogens detected by mNGS, and a “nonconformance” with final diagnosis was given to the mNGS detection. Further optimization of mNGS is required to obtain more reliable information for pathogen identification.

RNA-mNGS was performed in only 15 cases with suspected viral infection in this study. Viruses were detected in 14 out of the 15 cases, including 10 with RNA viruses (including 5 respiratory syncytial virus infections, 3 human rhinovirus infections, 1 influenza virus infection, and 1 parainfluenza infection). It seemed that RNA virus infection in children was common (66.67%, 10/15), and RNA-mNGS seemed to be a potential diagnostic tool. Due to the mild symptoms and self-limiting nature, viral respiratory infections are always ignored by clinicians. However, viral infections, especially novel viral infections, seem to have emerged more frequently in recent years. Thus, the viral profiles and their clinical characteristics in children with pneumonia need to be further explored.

In this study, the antibiotics of the 81 patients were reassessed based on their mNGS results. Except for 34 cases without treatment adjustment due to the coincidence of previous antibiotics with mNGS detection, as well as the consideration of self-limitation for some viruses, 58% of (47/81) patients received adjusted antibiotics. Finally, the conditions of most patients significantly improved. Our findings indicate that mNGS can not only accurately diagnose the pathogens causing pneumonia among children, but also has the potential to facilitate timely adjustment of treatments, thereby improving the prognosis of patients.

In conclusion, we evaluated the value of mNGS in the diagnosis of pneumonia in children. mNGS performed significantly better than conventional methods, especially when considering the limited volume of BALF and the use of empirical antibiotics. The rapid diagnosis of mNGS can facilitate timely adjustment of treatments, which has the potential to improve prognosis and decrease mortality.

## MATERIALS AND METHODS

### Ethics statement.

Study protocols were reviewed and approved by the Ethical Review Committee of the Guangdong Provincial People’s Hospital and Guangzhou Women and Children’s Medical Center (approval number KY-Q-2021-307-01). All procedures followed were in accordance with the ethical standards of the responsible committees on human experimentation (institutional and national) and with the Helsinki Declaration of 1975, as revised in 2000. Informed consents were obtained from the guardians of all patients enrolled in the study.

### Samples collection.

A total of 115 children with suspected pneumonia, who were admitted to Guangdong Provincial People’s Hospital and Guangzhou Women and Children’s Medical Center from February 2019 to January 2021, were enrolled in this study. The enrollment criteria were as follows: (i) pulmonary infection was considered with unfavorable clinical outcomes manifesting as unrelieved symptoms of fever and expectoration, or the lesions failing to absorb or even progressing based on reexamination of chest imaging, and the etiology needed to be clarified; (ii) chest imaging showed abnormalities, including tracheal and bronchopulmonary dysplasia or malformation, atelectasis, emphysema, pulmonary mass, and diffuse pulmonary disease; (iii) patients with difficulty in weaning from ventilator, and BALF was considered; and (iv) BAL was conducted and BALF was sent for mNGS detection with parental consent. Children with the following clinical manifestations were excluded from this study: (i) severe cardiopulmonary hypofunction, unstable circulation, and intolerance to the examination; (ii) active massive hemoptysis, severe bleeding disorders, and coagulation disorders; (iii) recent history of pulmonary surgery; and (iv) refusal to perform BAL. BALF of all children was collected according to Chinese Guidelines for Pediatric Flexible Bronchoscopy (2018) (38). Sputum, throat swabs, and blood of each patient were also collected at the same time.

### Conventional methods detection.

All the above samples were subjected to a series of laboratory tests immediately. Concretely, conventional methods, including cultures of BALF and sputum, smear, PCR of blood and throat swabs (including parvovirus, herpes simplex virus, human gammaherpesvirus 4, and human betaherpesvirus 5; SARS-CoV-2; respiratory syncytial virus; influenza A/B virus; parainfluenza virus; adenovirus; Mycoplasma pneumoniae; and Chlamydia pneumoniae), and serology tests of blood (G test, GM test, and M. pneumoniae antibody detection) were performed in these cases.

### mNGS detection using BALF samples.

DNA of each BALF sample (~1 mL) was extracted using QIAamp DNA Micro kit (Qiagen, Hilden, Germany) according to its manual. Total RNA of 15 BALF samples was extracted using QIAamp Viral RNA kit (Qiagen, Hilden, Germany), followed by removal of rRNA using Ribo-Zero rRNA Removal kit (Illumina, San Diego, USA) according to the instructions. cDNA was generated using reverse transcriptase and dNTPs (Thermo Fisher Scientific, MA, USA). The libraries (DNA and cDNA) were then built using QIAseq Ultralow Input Library kit for Illumina (Qiagen, Hilden, Germany). The quality of each library was assessed using Qubit (Thermo Fisher Scientific, MA, USA) and Agilent 2100 Bioanalyzer (Agilent Technologies, Palo Alto, USA). The qualified libraries were finally sequenced on a Nextseq 550 platform (Illumina, San Diego, USA) with 75-bp single-end reads for approximately 20 million per library after sequencing.

For bioinformatics analysis, adapter sequences as well as short (length < 36 bp), low-quality (Q < 30), and low-complexity reads were first filtered out from the raw data using bowtie2, followed by human host DNA reads after mapping to human reference database (hg38). The remaining reads were finally aligned to the Microbial Genome Databases (ftp://ftp.ncbi.nlm.nih.gov/genomes/) using Burrows-Wheeler Aligner. Least common ancestor was used for the species annotation analysis. Negative controls (NTC; sterile deionized water) and positive controls (synthesized fragments with known quantities) were established for each batch of experiments using the same wet lab procedures and bioinformatics analysis as the clinical samples. The positive criteria of mNGS were as follows: for bacteria (excluding Mycobacterium, Nocardia, and Legionella pneumophila), fungi, virus, and parasites, the result was considered positive if at least 3 nonoverlapping reads were mapped to species level and absent in NTC or the detected reads were ≥10-fold than that in the NTC. For Mycobacterium, *Nocardia*, and Legionella pneumophila, the result was considered positive if at least one species-specific read was detected, or the detected reads were ≥5-fold than that in the NTC.

### Statistical analysis.

Chi-square test and Fisher exact test were calculated to examine the differences of detection results between mNGS and conventional methods.

### Data availability.

The data sets used and/or analyzed during the current study are available at National Genomics Data Center (http://ngdc.cncb.ac.cn), reference number PRJCA008730.
